# 3,6-Carbazole vs 2,7-carbazole: A comparative study of hole-transporting polymeric materials for inorganic–organic hybrid perovskite solar cells

**DOI:** 10.3762/bjoc.12.134

**Published:** 2016-07-07

**Authors:** Wei Li, Munechika Otsuka, Takehito Kato, Yang Wang, Takehiko Mori, Tsuyoshi Michinobu

**Affiliations:** 1 Department of Materials Science and Engineering, Tokyo Institute of Technology, 2-12-1 Ookayama, Meguro-ku, Tokyo 152-8552, Japan and; 2 Department of Mechanical Engineering, National Institute of Technology, Oyama College, 771 Nakakuki, Oyama, Tochigi 323-0806, Japan

**Keywords:** carbazole polymer, hole transport, perovskite solar cell, polycondensation

## Abstract

The ever increasing demand for clean energy has encouraged researchers to intensively investigate environmentally friendly photovoltaic devices. Inorganic–organic hybrid perovskite solar cells (PSCs) are very promising due to their potentials of easy fabrication processes and high power conversion efficiencies (PCEs). Designing hole-transporting materials (HTMs) is one of the key factors in achieving the high PCEs of PSCs. We now report the synthesis of two types of carbazole-based polymers, namely 3,6-Cbz-EDOT and 2,7-Cbz-EDOT, by Stille polycondensation. Despite the same chemical composition, 3,6-Cbz-EDOT and 2,7-Cbz-EDOT displayed different optical and electrochemical properties due to the different connectivity mode of the carbazole unit. Therefore, their performances as hole-transporting polymeric materials in the PSCs were also different. The device based on 2,7-Cbz-EDOT showed better photovoltaic properties with the PCE of 4.47% than that based on 3,6-Cbz-EDOT. This could be due to its more suitable highest occupied molecular orbital (HOMO) level and higher hole mobility.

## Introduction

Inorganic–organic hybrid perovskite solar cells (PSCs) have recently received significant attention due to their remarkably high power convention efficiencies (PCEs). After the seminal study reported by Miyasaka et al. in 2009 with the PCE of 4% [1], some key improvements have been made in designing device structures and fabrication methods, and the PCE of the PSCs rapidly increased to >20% [2–5]. Compared to the conventional organic photovoltaics and dye-sensitized solar cells (DSSCs), PSCs benefit from a broad light absorption and high carrier diffusion length as excellent features of the perovskite materials. The development of efficient hole-transporting materials (HTMs), which extract a hole from the perovskite layer and transport it to the anode, is also significant for the further improvement of the PSC performances. So far, 2,2’,7,7’-tetrakis(*N*,*N*-di(*p*-methoxyphenyl)amino)-9,9-spirobifluorene (spiro-OMeTAD) is regarded as the most conventional solid state hole transporter for the PSCs [6–10]. Despite the high PCEs conferred by this hole transporting layer, spiro-OMeTAD has several limitations, such as its complicated multistep synthesis, low hole mobility in its pristine form, and expensive fabrication costs of the PSCs due to the sublimable small molecule. Accordingly, solution-processable hole-transporting polymers with simple structures have also been pursued as HTMs in the PSCs. Common p-type semiconducting polymers, such as poly(3-hexylthiophene) (P3HT), and the state-of-the-art narrow band gap polymers, such as poly[2,5-bis(2-decyldodecyl)pyrrolo[3,4-*c*]pyrrole-1,4(2*H*,5*H*)-dione-(*E*)-1,2-di(2,2’-bithiophen-5-yl)ethane (PDPPDBTE), were successfully applied to the HTM in the PSCs [11–12]. Among them, the poly(triarylamine) derivatives are currently some of the best polymeric hole-transporting materials for the PSCs [13].

Carbazole (Cbz)-based conjugated polymers are widely used as active photo- and semiconducting materials in a variety of organic electronics due to their tunable optical and electrical properties [14–21]. For example, the 3,6-positions of the carbazole readily react with various electrophiles, and accordingly, many types of linear and hyperbranched poly(3,6-carbazole) derivatives have been reported to show potent redox activities and nonlinear optical or photorefractive properties [22–24]. These features were also applied to organic light-emitting diodes (OLEDs) [25]. In contrast, the poly(2,7-carbazole) derivatives appeared after the pioneering synthetic studies of the 2,7-dihalogeno-carbazole monomers [26–27]. A series of 2,7-carbazole-based copolymers were synthesized, and most of them showed better semiconducting properties in organic field effect transistors (OFETs), bulk-heterojunction (BHJ) solar cells, thermoelectric and electrical memory devices as compared to the counter 3,6-carbazole-based polymers [28–36]. However, this structural relationship between the 3,6-carbazole and 2,7-carbazole has not yet been eluciated in the PSCs because of the emerging new devices.

In this study, we designed two carbazole-based hole-transporting polymers with different connectivity patterns. In order to enhance the electron-donating properties, the carbazole monomers were copolymerized with the electron-rich 3,4-ethylenedioxythiophene (EDOT) unit by Stille polycondensation. The energy levels and hole mobilities of the resulting carbazole copolymers, namely 3,6-Cbz-EDOT and 2,7-Cbz-EDOT, were estimated from the optical and electrochemical measurements and OFET performances, respectively. Finally, they were applied as the hole-transporting layer of the PSCs, and the photovoltaic properties of both devices were compared. The device based on 2,7-Cbz-EDOT displayed a higher PCE of 4.47% than that based on 3,6-Cbz-EDOT, which could be explained by their energy levels and hole mobilities.

## Results and Discussion

### Polymer synthesis

**Scheme 1:**
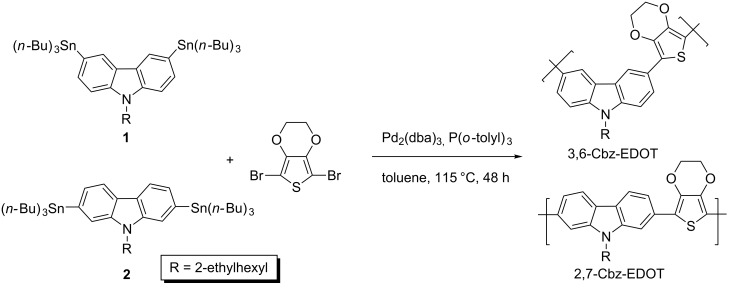
Synthesis of 3,6-Cbz-EDOT and 2,7-Cbz-EDOT by Stille polycondensation.

Carbazole copolymers with different connectivity patterns were synthesized by the Stille polycondensation between the bis(tri-*n*-butylstannyl)carbazole monomers and 2,5-dibromo-EDOT (Scheme 1). We very recently reported the synthesis of the same polymer structures by the microwave-assisted direct arylation polycondensation, but it should be noted that the soluble 2,7-Cbz-EDOT could not be obtained due to undesired side reactions [37]. In contrast, the conventional Stille polycondensation afforded the desired linear polymers without any side reactions. The number-averaged molecular weight (*M*_n_) and polydispersity indices (*M*_w_/*M*_n_), determined by GPC using THF as the eluent, were 2.0 kg mol^−1^ (*n* ~ 5) and 1.5 for 3,6-Cbz-EDOT and 3.0 kg mol^−1^ (*n* ~ 7) and 1.4 for 2,7-Cbz-EDOT, respectively. The polymer structures were characterized by ^1^H NMR and FTIR spectroscopies (Figure S1, Supporting Information File 1). The ^1^H NMR spectra revealed the ethylene groups of EDOT at ca. 4.2 ppm and the methylene group directly attached to the nitrogen atom of the carbazole at ca. 4.4 ppm, respectively, suggesting the successful copolymerization. Both polymers showed an explicit difference in their chemical shift values ascribed to the carbazole units due to the different connectivity patterns. In contrast, the IR spectra of the polymers were almost the same.

### Optical and electrochemical properties

**Figure 1:**
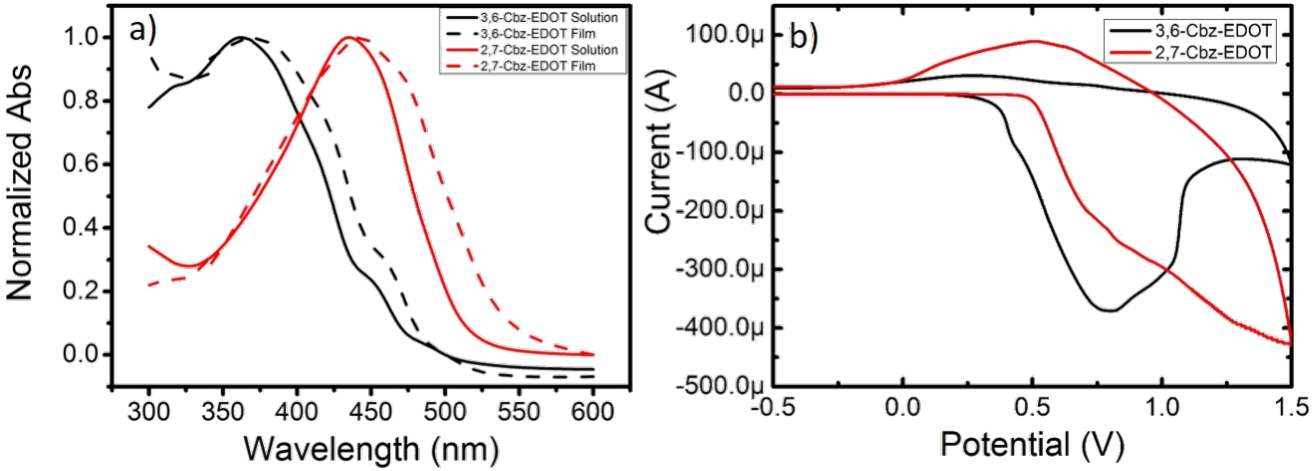
(a) Normalized UV–vis absorption of Cbz-EDOT polymers in CH_2_Cl_2_ measured at 10^−5^ M repeat unit^−1^ and in thin films and (b) cyclic voltammetry of Cbz-EDOT polymer films on glassy carbon electrode, measured in CH_3_CN with 0.1 M (*n*-C_4_H_9_)_4_NPF_6_ at the scan rate of 0.1 V s^−1^ under flowing nitrogen.

The UV–vis absorption spectra were employed to evaluate the optical properties of 3,6-Cbz-EDOT and 2,7-Cbz-EDOT. The spectra were measured in CH_2_Cl_2_ and in thin films (spin-coated on an ITO glass). The absorption maximum (λ_max_) of 3,6-Cbz-EDOT in CH_2_Cl_2_ was 362 nm, whereas the thin film showed the bathochromically-shifted λ_max_ at 374 nm (Figure 1a). Compared to 3,6-Cbz-EDOT, 2,7-Cbz-EDOT showed a lower energy absorption in both the solution and thin film states (λ_max_ = 435 nm in CH_2_Cl_2_; λ_max_ = 443 nm in the thin film), which is consistent with previous reports [26]. The red shifts in the absorption spectra from the solution to thin film states implied the presence of strong intermolecular interactions between the polymer backbones.

Cyclic voltammetry (CV) of the polymer thin films was measured in CH_3_CN with 0.1 M (*n-*C_4_H_9_)_4_NPF_6_ as the supporting electrolyte at 20 °C. Both polymers displayed irreversible oxidation peaks in the measurement range (Figure 1b). 3,6-Cbz-EDOT exhibited the onset oxidation potential (*E*_ox,onset_) at 0.38 V (vs Ag/Ag^+^), whereas the *E*_ox,onset_ of 2,7-Cbz-EDOT was slightly higher (0.50 V). This difference can also be explained by the different connectivity pattern between the two polymers. The linkage through the 3,6-positions of the carbazole unit forms a linear conjugation between the nitrogen atoms, which facilitates the electron removal from the conjugated main chain. In contrast, the nitrogen atoms through the 2,7-linked carbazoles are classified as a formal cross-conjugated structure [38].

As an efficient HTM for the PSCs, p-type polymers should have a good energy balance with the perovskite layer [39]. Most importantly, the highest occupied molecular orbital (HOMO) energy level should be close to the valence band of the perovskite. The HOMO levels of 3,6-Cbz-EDOT and 2,7-Cbz-EDOT were calculated according to Equation 1:

[1]
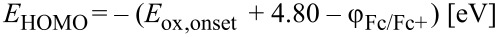


where φ_Fc/Fc+_ is the redox potential of the ferrocene/ferrocenium (Fc/Fc^+^) couple measured under the same conditions (in this case, 0.09 V vs the Ag/Ag^+^electrode). 2,7-Cbz-EDOT has a deeper HOMO level than 3,6-Cbz-EDOT. This was also the case for the lowest unoccupied molecular orbital (LUMO) levels, which were calculated from the HOMO levels and optical band gaps. All the data are listed in Table 1.

**Table 1:** Optical, electrochemical, and electrical properties of Cbz-EDOT polymers^a^.

Polymer	λ_onset_ (nm)	Band gap (eV)^b^	*E*_ox,onset_ (V)^c^	HOMO (eV)^d^	LUMO (eV)^e^	Hole mobility (cm^2^ V^−1^ s^−1^)^f^

3,6-Cbz-EDOT	480	2.58	0.38	−5.09	−2.50	3.5 × 10^−7^
2,7-Cbz-EDOT	536	2.31	0.50	−5.21	−2.90	5.1 × 10^−6^

^a^In the thin film states. ^b^Calculated from the λ_onset_ (1240/λ_onset_). ^c^vs Ag/Ag^+ d^Calculated from *E*_ox,onset_ and φ_Fc/Fc+_. ^e^Calculated from the HOMO levels and optical band gaps. ^f^Determined from the OEFT performances.

The carrier mobilities of the HTMs are some of the important parameters that determine the PSC performances. In order to elucidate the charge-transporting properties of the Cbz-EDOT polymers, top-contact/bottom-gate type OFET devices were fabricated, and the transistor performances were initially evaluated in air (for details see Supporting Information File 1). Both Cbz-EDOT polymers showed a p-type unipolar behavior during the measurements (Figure S2, Supporting Information File 1). Although the hole mobilities of both polymers were disappointedly low (<10^−5^ cm^2^ V^−1^ s^−1^) probably due to the unoptimized annealing conditions, the data clearly suggested that 2,7-Cbz-EDOT has a hole mobilitiy one-order higher than 3,6-Cbz-EDOT (Table 1). This result is also consistent with the reported mobilities of other carbazole-based semiconducting polymers [40–41].

### Photovoltaic performances

**Figure 2:**
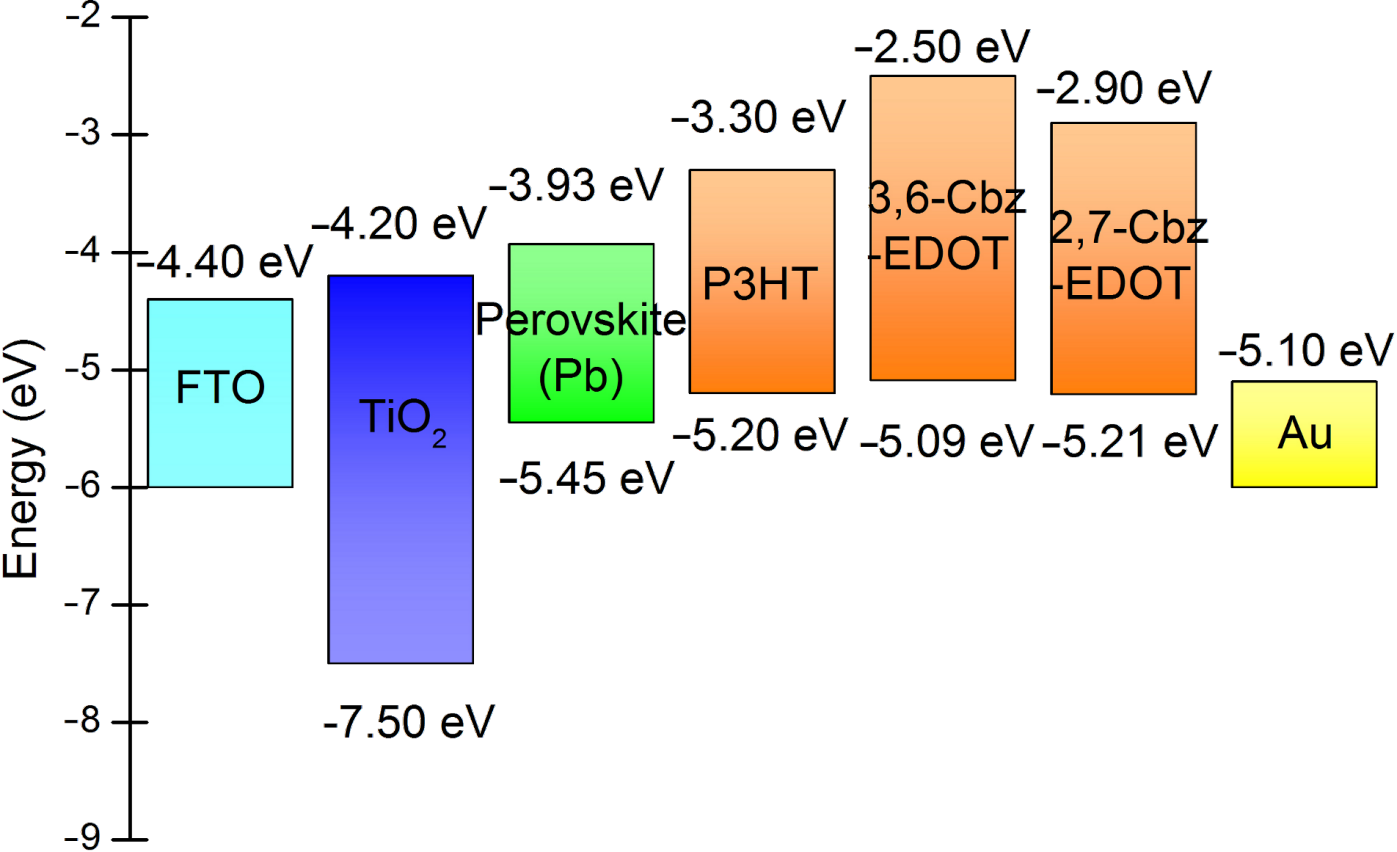
Energy level diagram of PSC components including P3HT, 3,6-Cbz-EDOT, and 2,7-Cbz-EDOT.

It was shown that the HOMO level (−5.21 eV) of 2,7-Cbz-EDOT is deeper than that (−5.09 eV) of 3,6-Cbz-EDOT. The former value is comparable to that (−5.20 eV) of P3HT and closer to the valence band (−5.45 eV) of the perovskite layer employed in this study. Accordingly, this carbazole polymer (2,7-Cbz-EDOT) is expected to be a better HTM in the PSCs as compared to 3,6-Cbz-EDOT. In order to evaluate this theory, PSCs with the Cbz-EDOT hole-transporting layer were fabricated, and the photovoltaic properties were compared. The device based on P3HT was also fabricated as the control sample [42]. The device structure is FTO/TiO_2_/perovskite (CH_3_NH_3_PbI_3_)/Cbz-EDOT (or P3HT)/Au, and the energy level diagram of the materials used in the devices is depicted in Figure 2.

**Figure 3:**
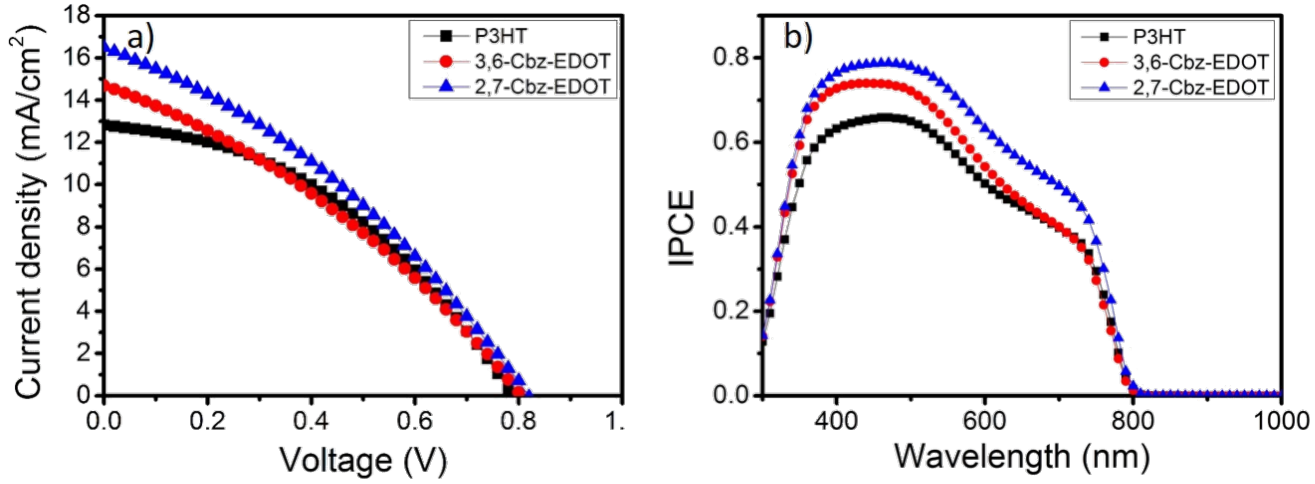
(a) Current density–voltage curves and (b) incident photon to current conversion efficiency (IPCE) spectra for PSCs with different HTMs under AM1.5G illumination at 100 mW cm^−2^.

Figure 3a shows the current density–voltage (*J*–*V*) curves for the devices based on 3,6-Cbz-EDOT, 2,7-Cbz-EDOT, and P3HT. The device performance parameters are summarized in Table 2. Among the three devices, the device based on 2,7-Cbz-EDOT displayed the highest power conversion efficiency (PCE) of 4.47% with a short-circuit current density (*J*_SC_) of 16.5 mA cm^−2^, open circuit voltage (*V*_OC_) of 0.81 V, and fill factor (FF) of 0.33. The lower PCE (3.90%) of the device based on 3,6-Cbz-EDOT was mainly due to the lower *J*_SC_ (14.7 mA cm^−2^), which would be reflected in the absorption range and hole mobility of the HTMs [43]. In addition, it was postulated that the deeper HOMO level of 2,7-Cbz-EDOT facilitated the hole extraction from the valence band of the perovskite layer [44]. Moreover, it should be noted that 2,7-Cbz-EDOT outperforms the benchmark p-type semiconducting polymer, P3HT, despite almost the same HOMO levels.

**Table 2:** Photovoltaic parameters of PSCs based on 3,6-Cbz-EDOT, 2,7-Cbz-EDOT, and P3HT.

HTM	*J*_sc_ (mA/cm^2^)^a^	*V*_oc_ (V)^a^	FF^a^	PCE (%)^a^	*R*_s_ (Ω)^b^	τ (μs)^b^

3,6-Cbz-EDOT	14.7	0.80	0.32	3.90	26.51	3.98
2,7-Cbz-EDOT	16.5	0.81	0.33	4.47	25.98	2.51
P3HT	12.8	0.79	0.41	4.14	17.94	1.99

^a^Average values from 10 devices with the sample area of 0.75 cm^2^ under illumination of 100 mW cm^−2^. ^b^Average values from 10 devices with the DC potential of 0 V, AC amplitude of 10 mV, and frequency of 1 Hz to 1 MHz under illumination of 100 mW cm^−2^.

The incident photon to current conversion efficiency (IPCE) spectra of the three devices were measured (Figure 3b). The perovskite nanocrystals strongly absorbed light over the entire visible range, and accordingly, the IPCE spectral shapes of all the devices were almost identical. This result indicates that light absorption by the hole-transporting polymers does not significantly contribute to the photocurrent generation and the main role of the polymers is hole transport. The peak intensities of the IPCE spectra obtained from the devices based on 3,6-Cbz-EDOT and 2,7-Cbz-EDOT exceeded 0.70 in the wavelength range of 380–510 nm. This value is apparently higher than that of the device based on P3HT with the maximum IPCE peak intensity of 0.66 at 470 nm, as supported by the *J*_SC_ values obtained from the *J*–*V* curves. All these results strongly suggest that the Cbz-EDOT polymers are better HTMs in the PSCs as compared to P3HT.

### Dynamic impedance spectroscopy

**Figure 4:**
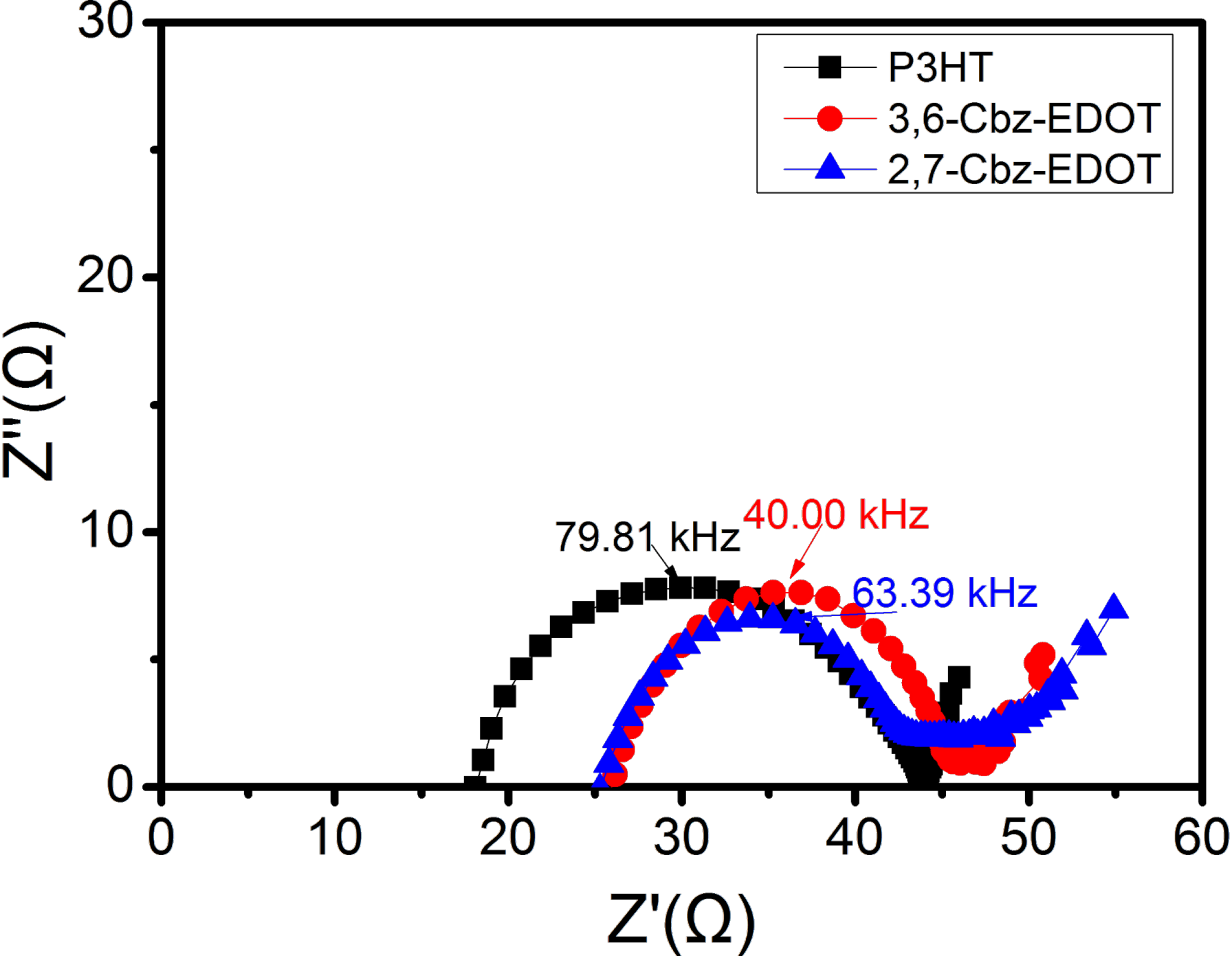
Impedance spectroscopy characterization of the PSCs with different HTMs over the frequency range from 1 Hz to 1 MHz at 0 V bias voltage under simulated AM1.5G illumination (100 mW cm^−2^).

Dynamic impedance spectroscopy was used to determine the charge-transporting parameters in the PSCs, such as the chemical capacitance, recombination resistance, and charge conductivity [45]. It is known that the performance metrics of the HTMs in the PSCs can be partially explained by these parameters. For example, it was suggested that FF is associated with the series resistance (*R*_s_), and a low *R*_s_ is required to construct high performance photovoltaic devices [46]. The dynamic impedance spectra of the PSCs with different HTMs over the frequency range of 1 Hz to 1 MHz are shown in Figure 4, and the *R*_s_ values are listed in Table 2. It was found that the order of *R*_s_ is *R*_s_(P3HT) < *R*_s_(2,7-Cbz-EDOT) < *R*_s_(3,6-Cbz-EDOT), which is in good agreement with the inverse order of the FFs of the corresponding PSCs (vide supra).

On the other hand, the effective carrier lifetime or time constant (τ) is related to the recombination of an electron and a hole at the interfaces between the perovskite and TiO_2_ or hole-transporting layers. This parameter can be calculated according to Equation 2 [47]:

[2]
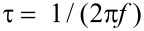


where *f* is the maximum frequency derived from the semicircle of the impedance spectra, also known as the character frequency. The calculated τ values are listed in Table 2. The device based on 3,6-Cbz-EDOT displayed the longest lifetime of 3.98 μs, which is almost twice as high as that of the device based on P3HT. This result can be explained by the LUMO levels of the HTMs [48]. Since 3,6-Cbz-EDOT has the highest LUMO level of −2.50 eV, the electron-blocking ability of this hole-transporting layer is more significant than the other two polymer layers. There is indeed a clear correlation between the τ values of the devices and the LUMO levels of the HTMs.

## Conclusion

In summary, highly electron-rich donor–donor type polymers with the different carbazole connectivity patterns, i.e., 3,6-Cbz-EDOT and 2,7-Cbz-EDOT, were synthesized by the Pd-catalyzed Stille polycondensation. The HOMO/LUMO energy levels of these polymers were determined from the onset oxidation potentials and optical band gaps. Similar to the reported carbazole-based semiconducting polymers, 3,6-Cbz-EDOT had higher energy levels than 2,7-Cbz-EDOT. These Cbz-EDOT polymers were applied to the hole-transporting layer of PSCs, and the photovoltaic properties were investigated in comparison to that based on P3HT. The 2,7-Cbz-EDOT based device showed the higher PCE of 4.47% as compared to those based on 3,6-Cbz-EDOT and also P3HT. This was due to the deeper HOMO level and higher hole mobility, suggesting the importance of the molecular design regarding the carbazole connectivity. In addition, the IPCE spectra suggested the higher photocurrent generation of the device based on 2,7-Cbz-EDOT, which is associated with the *J*_SC_. Furthermore, the impedance spectroscopy characterization revealed that the carrier recombination of the devices based on the Cbz-EDOT polymers is effectively suppressed due to the prolonged carrier lifetime. It is worth noting that there is still a room for improvement of the carrier mobility and lifetime by the increase in the molecular weights of these polymers. Overall, the Cbz-EDOT polymers, especially 2,7-Cbz-EDOT, are solution-processable promising HTMs of the PSCs.

## Experimental

### Materials

3,6-Dibromo-9-(2-ethylhexyl)carbazole [49] and 2,7-dibromo-9-(2-ethylhexyl)carbazole [27] were prepared according to the reported literature procedure. Commercially available solvents and reagents were used without further purification unless stated otherwise. P3HT (regioregular) was purchased from Aldrich.

### Synthesis

#### Synthesis of 9-(2-ethylhexyl)-3,6-bis(tri-*n*-butylstannyl)carbazole (**1**)

To a solution of 3,6-dibromo-9-(2-ethylhexyl)carbazole (0.360 g, 0.800 mmol) in THF (10 mL), *n*-butyllithium (1.6 M, 1.20 mL) in THF was added dropwise at −78 °C under Ar. After stirring for 30 min, 0.88 mL of chlorotributyltin (1.04 g, 2.00 mmol) were injected by a syringe. Then, the mixture was warmed to 0 °C and stirred at this temperature for 30 min. After the mixture was further warmed and stirred at room temperature overnight, water was added and the organic layer was extracted with CH_2_Cl_2_ (3 times). The combined organic layer was dried over MgSO_4_. After filtration, the solvent was removed under reduced pressure and the crude product was purified by column chromatography (SiO_2_, CHCl_3_), yielding the desired product **1** (0.338 g, 48%). ^1^H NMR (300 MHz, CDCl_3_) δ 8.22 (s, 2H), 7.55 (d, *J* = 7.9 Hz, 2H), 7.42 (d, *J* = 8.0 Hz, 2H), 4.17 (dd, *J* = 7.2, 3.8 Hz, 2H), 2.10 (s, 1H), 1.64 (d, *J* = 7.6 Hz, 12H), 1.42 (dd, *J* = 14.6, 7.3 Hz, 21H), 1.26–1.09 (m, 12H), 0.95 ppm (dd, *J* = 8.4, 6.2 Hz, 25H); ^13^C NMR (75 MHz, CDCl_3_) δ 140.85, 133.09, 129.25, 128.03, 122.81, 108.78, 47.15, 39.37, 30.86, 29.07, 28.74, 27.71, 27.32, 26.96, 26.73, 24.27, 22.96, 17.37, 13.94, 13.58, 13.48, 10.75, 9.59 ppm; FTIR (neat) ν: 2955, 2923, 2852, 2361, 1798, 1652, 1614, 1581, 1462, 1419, 1376, 1342, 1276, 1258, 1218, 1143, 1063, 1014, 961, 867, 794, 749, 698, 679, 654, 624, 607 cm^−1^; MALDI–TOF MS (*M*_w_ = 859.4): *m*/*z* = 860.7 ([M + H]^+^).

#### Synthesis of 9-(2-ethylhexyl)-2,7-bis(tri-*n*-butylstannyl)carbazole (**2**)

To a solution of 2,7-dibromo-9-(2-ethylhexyl)carbazole (0.437 g, 1.00 mmol) in THF (10 mL), *n*-butyllithium (1.6 M, 1.50 mL) in THF was added dropwise at −78 °C under Ar. After stirring for 30 min, 1.10 mL of chlorotributyltin (1.30 g, 2.50 mmol) was injected by a syringe. Then, the mixture was warmed to 0 °C and stirred at this temperature for 30 min. After the mixture was further warmed and stirred at room temperature overnight, water was added and the organic layer was extracted with CH_2_Cl_2_ (3 times). The combined organic layer was dried over MgSO_4_. After filtration, the solvent was removed under reduced pressure and the crude product was purified by column chromatography (SiO_2_, CHCl_3_), yielding the desired product **2** (0.407 g, 46%). ^1^H NMR (300 MHz, CDCl_3_) δ 8.05 (d, *J* = 7.5 Hz, 2H), 7.48 (s, 2H), 7.28 (d, *J* = 7.6 Hz, 2H), 4.18 (d, *J* = 6.5 Hz, 2H), 2.05 (s, 1H), 1.76–1.49 (m, 12H), 1.36 (dd, *J* = 14.7, 7.3 Hz, 21H), 1.12 (dd, *J* = 9.6, 6.7 Hz, 12H), 0.89 ppm (dd, *J* = 9.2, 5.3 Hz, 25H); ^13^C NMR (75 MHz, CDCl_3_) δ 140.90, 139.17, 126.57, 123.19, 119.98, 117.02, 47.31, 40.02, 31.53, 29.49, 27.73, 24.82, 23.38, 14.34, 14.00, 11.21, 10.08, 7.84 ppm; FTIR (neat) v: 2955, 2923, 2870, 2852, 1485, 1458, 1441, 1415, 1376, 1339, 1319, 1250, 1200, 1146, 1071, 996, 962, 911, 860, 826, 814, 794, 741, 723, 710, 695, 653, 633, 616 cm^−1^; MALDI–TOF MS (*M*_w_ = 859.4): *m*/*z* = 860.5 ([M + H]^+^).

#### Synthesis of 3,6-Cbz-EDOT

Analogously to the description in [50], a solution of **1** (0.337 g, 0.400 mmol) and 2,5-dibromo-3,4-ethylenedioxythiophene (0.117 g, 0.400 mmol) in toluene (5 mL) was degassed with nitrogen for 15 min. Pd_2_(dba)_3_ (0.010 g, 0.011 mmol) and P(*o*-tolyl)_3_ (0.015 g, 0.049 mmol) were added. The reaction mixture was further degassed and then heated to 110 °C for 48 h. After cooling to room temperature, the mixture was poured into methanol. The precipitate was collected and washed with methanol and hexane. The precipitate was subsequently subjected to Soxhlet extraction with chloroform. The solvent was removed under reduced pressure, yielding 3,6-Cbz-EDOT (0.10 g, 60%). GPC (THF): *M*_n_ = 2.0 kg mol^−1^, *M*_w_/*M*_n_ = 1.5; ^1^H NMR (300 MHz, CDCl_3_) δ 8.50–8.37 (br, Ar-H), 8.00–7.73 (br, Ar-H), 7.54–7.07 (br, Ar-H), 4.64–4.24 (br, CH_2_), 4.24–3.85 (br, OCH_2_), 2.04 (br, CH), 1.50–1.16 (br, CH_2_), 1.01–0.70 ppm (br, CH_3_); FTIR (neat) ν: 2955, 2921, 2852, 1581, 1463, 1419, 1375, 1341, 1276, 1258, 1219, 1142, 1063, 1015, 961, 868, 795, 749, 699, 653, 625, 607 cm^−1^.

#### Synthesis of 2,7-Cbz-EDOT

Analogously to the description in [50], a solution of **2** (0.392 g, 0.450 mmol) and 2,5-dibromo-3,4-ethylenedioxythiophene (0.136 g, 0.450 mmol) in toluene (5 mL) was degassed with nitrogen for 15 min. Pd_2_(dba)_3_ (0.010 g, 0.011 mmol) and P(*o*-tolyl)_3_ (0.015 g, 0.049 mmol) were added. The reaction mixture was further degassed and then heated to 110 °C for 48 h. After cooling to room temperature, the mixture was poured into methanol. The precipitate was collected and washed with methanol and hexane. The precipitate was subsequently subjected to Soxhlet extraction with chloroform. The solvent was removed under reduced pressure, yielding 2,7-Cbz-EDOT (0.13 g, 78%). GPC (THF): *M*_n_ = 3.0 kg mol^−1^, *M*_w_/*M*_n_ = 1.4; ^1^H NMR (300 MHz, CDCl_3_) δ 8.22–7.97 (br, Ar–H), 7.97–7.79 (br, Ar-H), 7.74–7.47 (br, Ar-H), 4.47 (br, CH_2_), 4.35–3.92 (br, O-CH_2_), 2.14 (br, CH), 1.62–1.17 (br, CH_2_), 1.12–0.67 ppm (br, CH_3_); FTIR (neat) ν: 2955, 2925, 2870, 1599, 1558, 1482, 1455, 1428, 1361, 1332, 1252, 1216, 1196, 1168, 1120, 1088, 997, 935, 906, 860, 801, 727, 720, 700, 675, 649, 633, 625, 618 cm^−1^.

### General measurements

^1^H NMR and ^13^C NMR spectra were measured on a JEOL model AL300 spectrometer at 20 °C. Chemical shifts are reported in ppm downfield from SiMe_4_, using the solvent’s residual signal as an internal reference. Fourier transform infrared (FTIR) spectra were recorded on a JASCO FT/IR-4100 spectrometer in the range from 4000 to 600 cm^−1^. MALDI–TOF mass spectra were measured on a Shimadzu/Kratos AXIMA-CFR mass spectrometer equipped with nitrogen laser (λ = 337 nm) and pulsed ion extraction, which was operated in a linear-positive ion mode at an accelerating potential of 20 kV. Dichloromethane solutions containing 1 g L^−1^ of a sample, 10 g L^−1^ of dithranol, and 1 g L^−1^ of sodium trifluoroacetate were mixed at the ratio of 1:1:1, and 1 mL aliquot of this mixture was deposited onto a sample target plate. Gel permeation chromatography (GPC) was measured on a JASCO GULLIVER 1500 equipped with a pump (PU-2080 Plus), an absorbance detector (RI-2031 Plus), and two Shodex GPC KF-803 columns (8.0 mm I.D. × 300 mm L) based on a conventional calibration curve using polystyrene standards. Tetrahydrofuran (40 °C) was used as a carrier solvent at the flow rate of 1.0 mL min^−1^. UV–vis absorption spectra were measured on a JASCO V-670 spectrophotometer. Polymer thin films were prepared on an ITO glass (about 0.8 × 2.5 cm^2^) by spray-coating of polymer solutions (5.0 g L^−1^ in CH_2_Cl_2_). Electrochemistry measurements were carried out on a BAS electrochemical analyzer model 612C at 25 °C in dehydrated CH_3_CN containing 0.1 M (*n*-C_4_H_9_)_4_NPF_6_ in the three electrode cell. The working, reference, and auxiliary electrodes were a glassy carbon electrode, Ag/Ag^+^/CH_3_CN/(*n*-C_4_H_9_)_4_NPF_6_, and a Pt wire, respectively.

### Fabrication and measurements of perovskite solar cells

Fluorine-doped tin oxide (FTO)-coated glass substrates (20 Ω per square) were patterned to fabricate the solar cells. These substrates were successively washed by ultrasonication in water, acetone, and isopropyl alcohol for 10 min each, and then dried in a stream of dry air. The washed substrates were further treated with a UV-O_3_ cleaner (Filgen, Model UV253E) for 20 min. The electron-accepting TiO_2_ compact layer was spin-coated (1500 rpm for 30 s) from a mildly acidic (after addition of 12 μM HCl) solution of titanium(IV) isopropoxide in anhydrous ethanol and sintered at 120 °C for 10 s. The mesoporous TiO_2_ layer composed of 20 nm-sized particles was deposited by spin-coating at 4000 rpm for 30 s using a commercial TiO_2_ paste (PST-18NR, JGC Catalysts and Chemicals Ltd.) diluted in ethanol (2:7, weight ratio). After drying at 120 °C, the TiO_2_ films were gradually heated to 500 °C, baked at this temperature for 20 min, and cooled to room temperature. The mesoporous TiO_2_ films were infiltrated with PbI_2_ by spin-coating a PbI_2_ solution in DMF (465 g L^−1^) at 80 °C. After drying, the films were dipped in a solution of CH_3_NH_3_I in 2-propanol (10 g L^−1^) for 20 s and rinsed with 2-propanol. After drying, the hole transporting materials were deposited by spin-coating a solution of P3HT, 3,6-Cbz-EDOT, or 2,7-Cbz-EDOT in chlorobenzene (7.82 g L^−1^). All hole-transporting layers were dried in the dark. The thickness of the hole-transporting layers was about 30 nm. Finally, gold (80 nm) was thermally evaporated on the top of the device to form the back contact.

The current density–voltage (*J*–*V*) characteristics, incident photon current efficiency (IPCE), and dynamic impedance spectroscopy of the fabricated perovskite solar cells were evaluated. The active area of the devices was 0.75 cm^2^. The *J*–*V* curves were measured on a CEP-2000RS (Bunko-Keiki Co., Ltd.) under illumination of a solar simulator with a light intensity of 100 mW cm^−2^ (AM1.5). IPCE measurements were carried out using a Xenon lamp attached to a monochromator. Two focusing lenses were used to focus the monochromatic light to the active area of solar cells. Dynamic impedance spectroscopy was measured on an SI1260 Impedance/Gain-Phase Analyzer and SI1286 Electrochemical Interface (Solartron) at 0 V bias voltage and a frequency range from 1 Hz and 1 MHz with an AC amplitude of 10 mA under illumination of simulated solar AM1.5 global light at 100 mW cm^−2^. A Z-View Analyst software was used to model the Nyquist plots obtained from the impedance measurements.

## Supporting Information

File 1 Synthesis of carbazole derivatives, ^1^H NMR and IR spectra of the polymers, and OFET performances.
