# Supplementary material for: 3,6-Carbazole vs 2,7-carbazole: A comparative study of hole-transporting polymeric materials for inorganic–organic hybrid perovskite solar cells
Source: Beilstein J Org Chem. 2016 Jul 7;12:1401–9. doi: 10.3762/bjoc.12.134 (PMC4979756; doi:10.3762/bjoc.12.134)
Supplement: File 1 — Synthesis of carbazole derivatives, 1H NMR and IR spectra of the polymers, and OFET performances. [file Beilstein_J_Org_Chem-12-1401-s001.pdf]

Supporting Information
for

3,6-Carbazole vs 2,7-carbazole: A comparative study of hole-transporting polymeric materials for inorganic–organic hybrid perovskite solar cells

Wei Li[1], Munechika Otsuka[2], Takehito Kato[2]*, Yang Wang[1], Takehiko Mori[1] and Tsuyoshi Michinobu[1]*

Address: [1]Department of Materials Science and Engineering, Tokyo Institute of Technology, 2-12-1 Ookayama, Meguro-ku, Tokyo 152-8552, Japan and [2]Department of Mechanical Engineering, National Institute of Technology, Oyama College, 771 Nakakuki, Oyama, Tochigi 323-0806, Japan

Email: Takehiko Kato - kato_t@oyama-ct.ac.jp; Tsuyoshi Michinobu - michinobu.t.aa@m.titech.ac.jp

* Corresponding authors

Synthesis of carbazole derivatives, ^{1}H NMR and IR spectra of the polymers, and OFET performances

Synthesis

Synthesis of 3,6-dibromo-9-(2-ethylhexyl)carbazole

Analogously to the description in [S1], a solution of 3,6-dibromocarbazole (1.00 g, 3.10 mmol) and NaH (0.20 g, 4.5 mmol) in DMF (5 mL) was degassed with nitrogen for 15 min. 2-Ethylhexyl bromide (0.90 g, 4.5 mmol) was added by a syringe. The mixture was stirred at room temperature for 24 h. Water was added, and the organic layer was extracted with CH_2Cl_2 (30 mL × 3). After the combined organic layers were dried over $MgSO_4$, the solvent was removed under reduced pressure. Column chromatography (SiO_2, hexane) gave the desired product as colorless liquid (1.00 g, 75%). ^{1}H NMR (300 MHz, $CDCl_3$): δ 8.08 (d, *J* = 1.7 Hz, 2H), 7.53 (dd, *J* = 1.8, 8.7 Hz, 2H), 7.20 (d, *J* = 8.7 Hz, 2H), 4.02 (d, *J* = 7.5 Hz, 2H), 2.15–1.83 (m, 1H), 1.41–1.08 (m, 8H), 1.00–0.69 ppm (m, 6H). ^{13}C NMR (75 MHz, $CDCl_3$): δ 140.49, 129.72, 124.12, 123.93, 112.64, 111.38, 48.23, 39.93, 31.54, 29.35, 24.93, 23.61, 14.61, 11.46 ppm. IR (neat): ν = 2958, 2928, 2869, 1847, 1719, 1622, 1588, 1548, 1470, 1437, 1380, 1363, 1343, 1285, 1147, 1057, 1018, 965, 911, 867, 834, 795, 742, 713, 665, 644, 614 cm^{-1}.

Synthesis of 2,7-dibromo-9-(2-ethylhexyl)carbazole

Analogously to the description in [S2], a solution of 2,7-dibromocarbazole (1.00 g, 3.10 mmol) and NaH (0.2 g, 4.5 mmol) in DMF (5 mL) was degassed with nitrogen for 15 min. 2-Ethylhexyl bromide (0.90 g, 4.5 mmol) was added by a syringe. The mixture was stirred at room temperature for 24 h. Water was added, and the organic layer was extracted with CH_2Cl_2 (30 mL × 3). After the combined organic layers were dried over $MgSO_4$, the solvent was removed under reduced pressure. Column chromatography (SiO_2, hexane) gave the desired product as colorless liquid (1.1 g, 82%). ^{1}H NMR (300 MHz, $CDCl_3$): δ 7.85 (d, *J* = 8.3 Hz, 2H), 7.48 (d, *J* = 1.1 Hz, 2H),

7.33 (dd, *J* = 1.4, 8.3 Hz, 2H), 4.00 (dd, *J* = 2.7, 7.5 Hz, 2H), 2.33–1.70 (m, 1H), 1.45–1.15 (m, 8H), 1.02–0.82 ppm (m, 6H). ^{13}C NMR (75 MHz, $CDCl_3$): δ 142.70, 123.35, 122.26, 122.07, 120.49, 113.12, 48.39, 39.84, 31.46, 29.23, 25.00, 23.72, 14.70, 11.56 ppm. IR (neat): ν = 2958, 2925, 2859, 1859, 1682, 1620, 1584, 1482, 1451, 1327, 1310, 1225, 1247, 1131, 1054, 997, 967, 946, 910, 876, 843, 820, 796, 767, 743, 701, 685, 666, 651, 638 cm^{-1}

^{1}H NMR and IR spectra

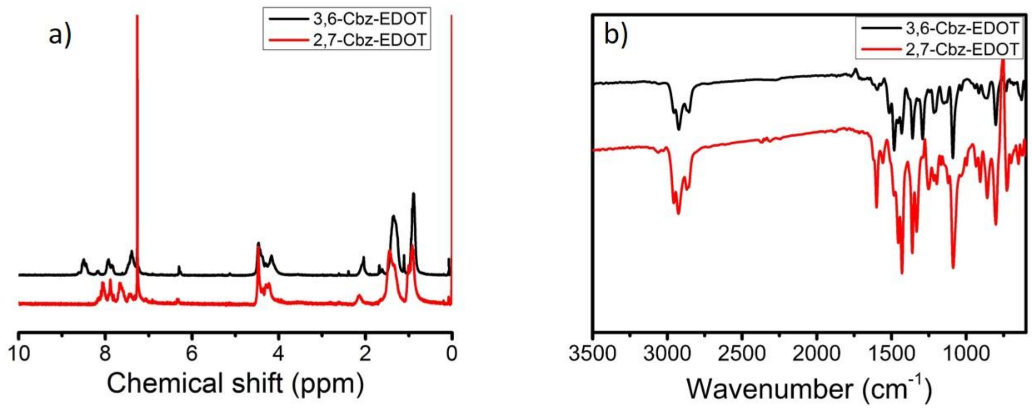

Figure S1: a) ^{1}H NMR spectra of 3,6-Cbz-EDOT and 2,7-Cbz-EDOT in $CDCl_3$ and b) IR spectra of 3,6-Cbz-EDOT and 2,7-Cbz-EDOT.

OFET performances

Top-contact/bottom-gate type organic field effect transistor (OFET) devices were fabricated using n^+-Si/SiO_2 substrates where n^+-Si and SiO_2 were used as the gate electrode and gate dielectric, respectively. After the substrates were washed by ultrasonication in acetone, 2-propanol, and deionized water, they were further cleaned by a UV-O_3 cleaner (Technovision, Model 208). The cleaned substrates were modified with hexamethyldisilazane (HMDS) to form a self-assembled monolayer (SAM). The polymer solutions in CH_2Cl_2 (7.0 g mL^{-1}) were spin-coated on the treated substrate to form the polymer thin films (40–60 nm thickness) in air. After that, 50 nm thick gold was deposited as the source and drain contacts using a shadow mask. The OFET devices had a channel length (L) of 100 μm and a channel width (W) of 1 mm. The OFET performances were measured in air using a Keithley 4200 parameter analyzer on a probe stage. The hole mobilities, μ, were calculated from the data in the saturated regime using Equation S1:

$$I_{SD} = (W/2L)C_i\mu(V_G-V_T)^2 \qquad \text{(S1)}$$

where I_{SD} is the drain current in the saturated regime, W and L are the semiconductor channel width and length, respectively, C_i (C_i = 13.7 nF cm^{-2}) is the capacitance per unit area of the gate dielectric layer, and V_G and V_T are the gate voltage and threshold voltage, respectively. $V_G - V_T$ of the devices was determined from the square root values of I_{SD} at the saturated regime.

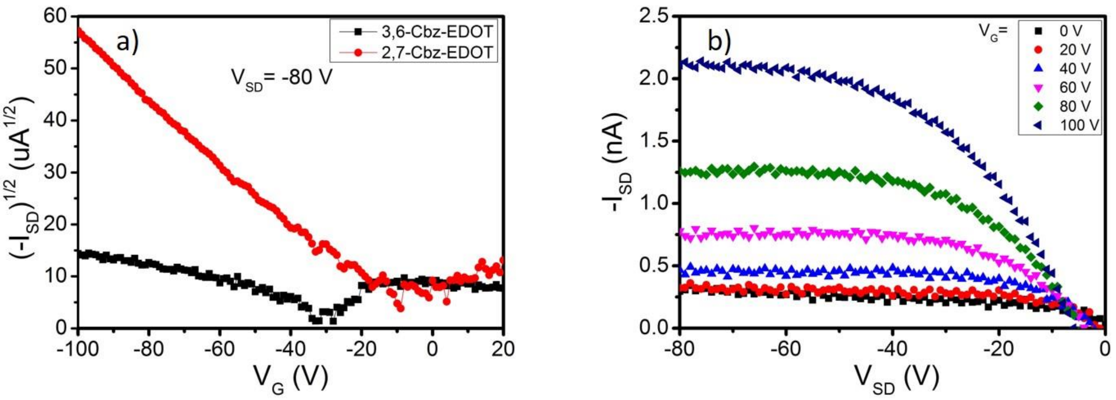

Figure S2: (a) Transfer characteristics of the OFETs based on 3,6-Cbz-EDOT and 2,7-Cbz-EDOT and (b) output characteristics of the OFET based on 2,7-Cbz-EDOT.

References

S1. Fujita, H.; Michinobu, T. *Macromol. Chem. Phys.*, **2012**, *213*, 447-457.

S2. Michinobu, T.; Osako, H.; Shigehara, K. *Macromolecules*, **2009**, *42*, 8172-8180.
